# Effects of Therapy with Fibrin Glue combined with Mesenchymal Stem Cells (MSCs) on Bone Regeneration: A Systematic Review

**DOI:** 10.3390/cells10092323

**Published:** 2021-09-05

**Authors:** Adriana de Cássia Ortiz, Simone Ortiz Moura Fideles, Karina Torres Pomini, Carlos Henrique Bertoni Reis, Cleuber Rodrigo de Souza Bueno, Eliana de Souza Bastos Mazuqueli Pereira, Jéssica de Oliveira Rossi, Paulo Cezar Novais, João Paulo Galletti Pilon, Geraldo Marco Rosa Junior, Daniela Vieira Buchaim, Rogerio Leone Buchaim

**Affiliations:** 1 Department of Biological Sciences, Bauru School of Dentistry (FOB/USP), University of São Paulo, Bauru 17012-901, SP, Brazil; adrianaortiz@usp.br (A.d.C.O.); simoneortiz@usp.br (S.O.M.F.); karinatp@usp.br (K.T.P.); dr.carloshenriquereis@usp.br (C.H.B.R.); cleuberbueno@usp.br (C.R.d.S.B.); jessicagoncalves@alumni.usp.br (J.d.O.R.); 2 Postgraduate Program in Structural and Functional Interactions in Rehabilitation, Postgraduate Department, University of Marilia (UNIMAR), Marília 17525-902, SP, Brazil; elianabastos@unimar.br (E.d.S.B.M.P.); paulonovais@unimar.br (P.C.N.); joao.pilon@unesp.br (J.P.G.P.); danibuchaim@alumni.usp.br (D.V.B.); 3 Pro-rectory of Graduate Studies and Research, Sacred Heart University Center (UNISAGRADO), Bauru 17011-160, SP, Brazil; geraldo.junior@unisagrado.edu.br; 4 Department of Human Anatomy and Neuroanatomy, University Center of Adamantina (UniFAI), Medical School, Adamantina 17800-000, SP, Brazil; 5 Graduate Program in Anatomy of Domestic and Wild Animals, Faculty of Veterinary Medicine and Animal Science, University of São Paulo (FMVZ/USP), São Paulo 05508-270, SP, Brazil

**Keywords:** bone regeneration, bone repair, fibrin glue, fibrin sealant, scaffolds, stem cells, systematic review

## Abstract

Cell therapy strategies using mesenchymal stem cells (MSCs) carried in fibrin glue have shown promising results in regenerative medicine. MSCs are crucial for tissue healing because they have angiogenic, anti-apoptotic and immunomodulatory properties, in addition to the ability to differentiate into several specialized cell lines. Fibrin sealant or fibrin glue is a natural polymer involved in the coagulation process. Fibrin glue provides a temporary structure that favors angiogenesis, extracellular matrix deposition and cell-matrix interactions. Additionally, fibrin glue maintains the local and paracrine functions of MSCs, providing tissue regeneration through less invasive clinical procedures. Thus, the objective of this systematic review was to assess the potential of fibrin glue combined with MSCs in bone or cartilage regeneration. The bibliographic search was performed in the PubMed/MEDLINE, LILACS and Embase databases, using the descriptors (“fibrin sealant” OR “fibrin glue”) AND “stem cells” AND “bone regeneration”, considering articles published until 2021. In this case, 12 preclinical and five clinical studies were selected to compose this review, according to the eligibility criteria. In preclinical studies, fibrin glue loaded with MSCs, alone or associated with bone substitute, significantly favored bone defects regeneration compared to scaffold without cells. Similarly, fibrin glue loaded with MSCs presented considerable potential to regenerate joint cartilage injuries and multiple bone fractures, with significant improvement in clinical parameters and absence of postoperative complications. Therefore, there is clear evidence in the literature that fibrin glue loaded with MSCs, alone or combined with bone substitute, is a promising strategy for treating lesions in bone or cartilaginous tissue.

## 1. Introduction

Advances in regenerative medicine have brought new therapeutic approaches to treat chronic injuries or to regenerate tissues with limited capacity for spontaneous regeneration, such as bone tissue [1]. Among the strategies commonly proposed to regenerate bone tissue, autogenous bone grafting is still considered the gold standard because it contains biological factors essential to tissue regeneration, such as osteogenic cells, cytokines, growth factors and extracellular matrix components [2,3,4]. However, some disadvantages are associated with this graft, such as the limited availability of bone, incidence of morbidity and pain. Likewise, the use of allogeneic bone graft also has certain limitations, such as the risk of infections and immune reactions, in addition to the loss of its biological properties due to the sterilization process [3,4]. Thus, cell therapy emerges as a segment of regenerative medicine that encompasses the use of cells, often combined with biomaterials, to regenerate damaged tissues in the organism [5,6].

**Figure 1:**
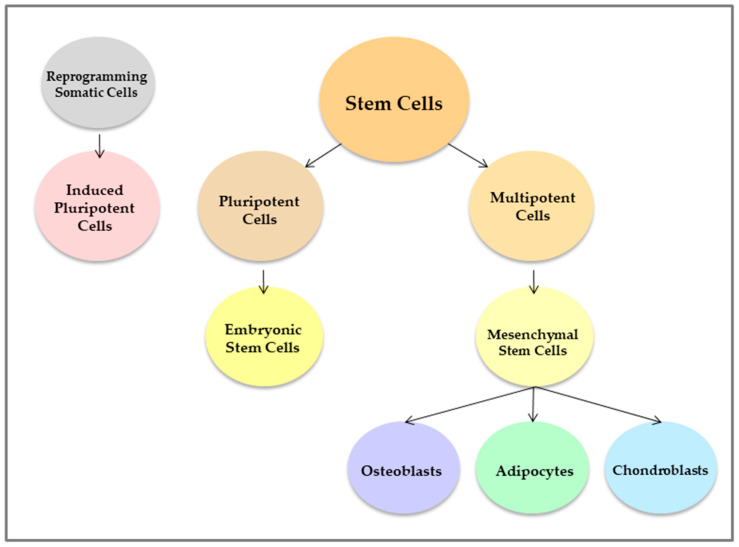
Stem cell lineages. Embryonic stem cells are pluripotent and differentiate into almost all specialized cell lines. In somatic cells, pluripotency can be induced through the regulation of transcription factors. Mesenchymal stem cells are multipotent stromal cells capable of differentiating into some specialized cell lines, such as osteoblasts, adipocytes and chondroblasts.

**Figure 2:**
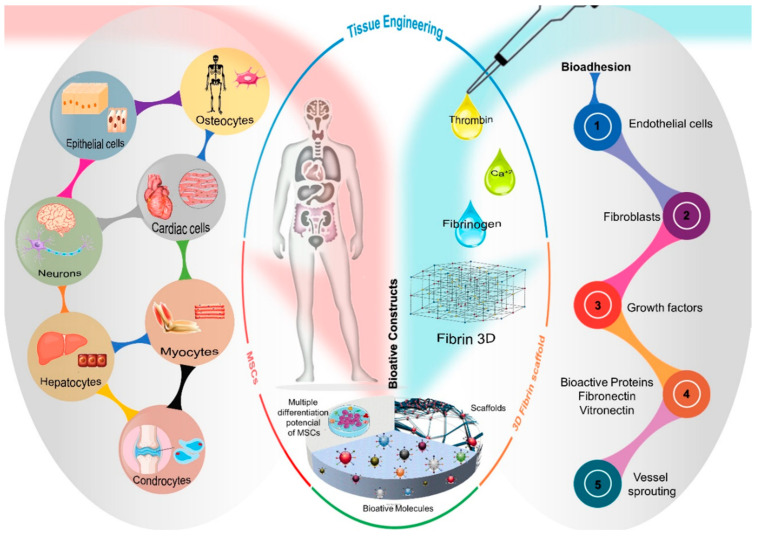
Schematic overview of cell types resulting from the MSCs differentiation process and the bioadhesion properties of the three-dimensional fibrin network structure, attracting the migration of endothelial cells, fibroblasts, vascular sprouts and biologically active molecules such as growth factors and proteins, *fibronectin* and *vitronectin*. Thus, tissue engineering uses the intrinsic characteristics of each biomaterial, such as the multiple differentiation potential of MSCs and the ability of the three-dimensional structure of fibrin to mimic a microenvironment favorable to cell growth, constituting a bioactive construct and, consequently, restoring morphofunctionality of the native tissue.

In cell therapy strategies, stem cells (SCs) have been used successfully for presenting characteristics that are essential to promote tissue regeneration, such as self-renewal capacity and potential for differentiation in specialized cell lines. Several SCs lineages have distinct differentiation potentials (Figure 1). Embryonic stem cells (ESCs) are pluripotent and have the ability to differentiate into most specialized cells in the organism. However, ethical issues limit the use of ESCs in clinical practice. To address this question, recent strategies have investigated the regenerative potential of induced pluripotent stem cells (iPSCs), obtained by reprogramming somatic cells through the regulation of transcription factors. However, the genetic manipulation of the cell may represent a limitation, so that researches have advanced in order to expand the use of this strategy. Other SCs lines are multipotent, such as mesenchymal stem cells (MSCs) [7,8]. MSCs are undifferentiated stromal cells present in most adult connective tissues and can be obtained from several sources, such as bone marrow, periosteum, adipose tissue, skin, muscle, tendons, umbilical cord, peripheral circulation and dental tissue [5,9,10]. MSCs are capable of self-renewal, proliferation and differentiation in some specialized cell lines, such as osteoblasts, chondroblasts and adipocytes (Figure 2) [5,7,8]. In addition, MSCs presents adherent growth on culture plastic and can be characterized by the expression of specific surface antigens, such as *CD90*, *CD73*, *CD29* and *CD105* [1,5,7,8].

MSCs have important biological properties and have been commonly used in transplants, tissue engineering and genetic therapies [5]. Due to their angiogenic, anti-apoptotic, immunomodulatory and immunosuppressive properties, MSCs play a fundamental role in tissue healing, modulating the production of important cytokines [7,8,9]. Through several signaling pathways, MSCs act by reducing the levels of inflammatory cytokines, such as *interleukin 6* (IL-6), *tumor necrosis factor-α* (TNF-α) and *interleukin 1β* (IL-1β), and favoring production of anti-inflammatory cytokines, such as *interleukin 10* (IL-10) and *interleukin 12* (IL-12). Increased *TNF-α* levels exacerbate the inflammatory response and favor osteoblast apoptosis [7,8,9]. Thus, the interactions that MSCs establish in the microenvironment control the inflammatory process and contribute to cell survival. MSCs also accelerate bone regeneration because they are able to migrate to the injury site and recruit osteogenic cells, through the release of chemotactic factors [7,10]. In addition, MSCs have angiogenic potential, favoring vascular neoformation through complex interactions with endothelial cells and the expression of angiogenic factors. Angiogenesis is a fundamental process for tissue regeneration, as it provides nutrients, oxygen, cells and growth factors to the injured area.

Considering the regenerative properties of MSCs, strategies have been proposed to deliver cells to the site of the injured tissue. In general, MSCs can be injected directly into the lesion or seeded into biocompatible scaffolds. Regarding bone tissue, the use of carrier biomaterials can accelerate bone formation, constituting the method of choice, especially in situations of extensive tissue damage. Several biomaterials have been proposed as cell carriers for the injured tissue [2]. Recently, a fibrin glue, also called fibrin sealant or fibrin tissue adhesive, has been used as a delivery system for cells, biomolecules, drugs and growth factors [11,12,13,14,15]. Fibrin is a natural polymer involved in the blood coagulation cascade and is formed by the action of *thrombin* on *fibrinogen* [13,16,17]. The use of allogeneic fibrin has been approved by the Federal Drug Administration (FDA) and has been used since 1976 as a hemostatic agent in coagulopathies and in various types of surgery, such as cardiovascular, thoracic, gastrointestinal, neurosurgery, among others [11,17,18]. In addition to acting as a hemostatic and healing agent, fibrin glue has certain advantages over other biomaterials when used as a delivery system [19].

Fibrin glue is biocompatible, non-cytotoxic and naturally biodegraded by the action of fibrinolytic enzymes [13,19,20,21]. Since it consists basically of *fibrinogen* and *factor XIII* (component I), *thrombin* and calcium chloride (component II), fibrin glue can be obtained from plasma components, enabling the construction of autologous scaffolds, which reduces the risk of infection and immunological reaction [11,17,22]. The fibrin glue also allows for a uniform distribution of cells in the scaffold and the implantation of this scaffold can be carried out by a less invasive technique that allows the filling of the lesion area [14,19]. In addition, fibrin glue constitutes a bioactive matrix that favors cell viability, adhesion, proliferation and differentiation [13,14,23]. The fibrin matrix contains binding sites with several cells and biomolecules that act in the tissue regeneration process, such as endothelial cells, fibroblasts, growth factors and several proteins in the extracellular matrix, such as *fibronectin* and *vitronectin* that regulate cell adhesion, migration and proliferation (Figure 2) [14,19]. Additionally, the fibrin matrix presents a porous structure that favors cell migration, vascular infiltration and the angiogenesis process, which are indispensable for tissue repair [13,14,23,24].

The biological properties of fibrin glue as a growth environment for MSCs have been reported in several studies [25,26,27,28]. In vitro studies that cultivated MSCs in fibrin glue showed that this natural polymer constitutes a favorable microenvironment for the survival, growth and maintenance of the paracrine functions of MSCs [25,26,27]. Kim et al. (2013) showed that MSCs grown in fibrin glue survived and secreted growth factors and immunomodulators, such as the *vascular endothelial growth factor, hepatocyte growth factor, transforming growth factor* and *prostaglandin*. In this study, when exposed to extreme stress, fibrin glue protected the MSCs from oxidative stress and prevented human dermal fibroblast death [26].

Several studies using animal models have shown the osteogenic potential of fibrin glue carried with MSCs. In these studies, the implantation of fibrin glue with MSCs isolated from bone marrow (BM-MSCs) resulted in ectopic bone formation [29,30], and no evidence of tumor growth was found in the implant area [29]. In combination with MSCs, fibrin glue has also been used successfully in several clinical applications such as reconstruction of bone defects, regeneration of injuries in cartilage, ligaments and tendons, regeneration of cardiac and peripheral nerve tissue, healing of severe burns and skin wounds, and treatment of chronic fistulas related to gastrointestinal disorders, such as Crohn’s disease [1,11].

Considering that fibrin glue associated with MSCs have interesting biological properties for use as a low-invasive cell therapy strategy to regenerate tissue injuries, the main objective of this systematic review was to search evidences in the literature about the regenerative potential of scaffolds containing fibrin glue loaded with MSCs in the fracture or bone defects and joint cartilage injuries healing.

## 2. Materials and Methods

### 2.1. Study Design and Bibliographic Search Strategy

**Figure 3:**
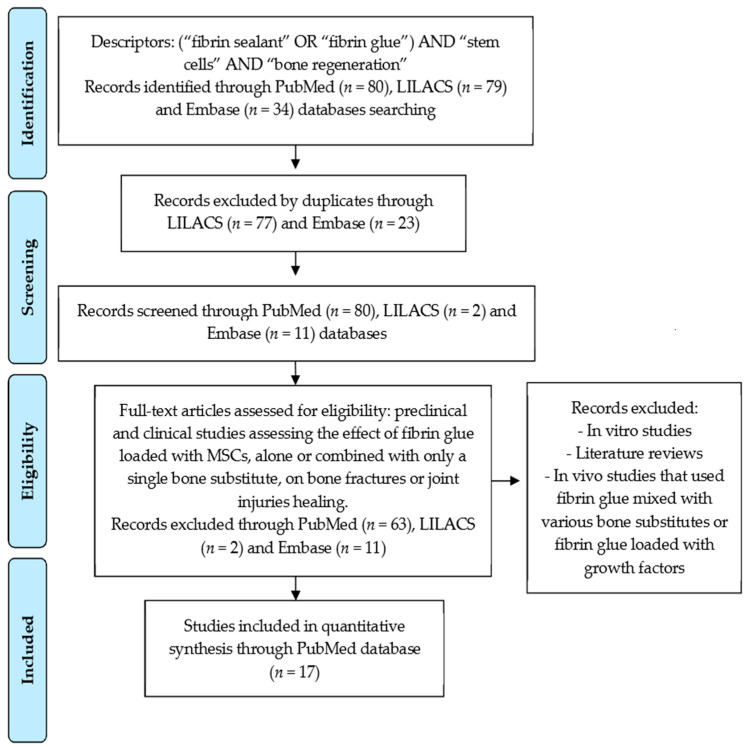
Prisma flow diagram resulted from the literature survey for the regenerative potential of fibrin glue loaded with MSCs on bone fractures and joint injuries healing.

This systematic review was carried out in April-May 2021 and was conducted in accordance with the PRISMA guidelines and the PICO strategy (Patient, Intervention, Comparison and Outcomes). The study design was structured in the selection of clinical trials and preclinical studies that used animal models to evaluate the regenerative potential of fibrin glue loaded with MSCs in the healing of fractures, bone defects and cartilage injuries. The electronic bibliographic search was carried out in the PubMed/MEDLINE, Embase and LILACS databases, combining the descriptors (“fibrin sealant” OR “fibrin glue”) AND “stem cells” AND “bone regeneration”. All studies published until the year 2021 were considered. The results of the bibliographic search are described in the Prisma Flow Diagram (Figure 3).

### 2.2. Study Eligibility

The eligibility criteria included clinical studies that evaluated the effect of fibrin glue combined with MSCs on bone fractures or joint injuries healing. In vivo studies that used animal models to investigate the potential of fibrin glue loaded with MSCs to regenerate bone defects or cartilage injuries were also included in this review. In vitro studies, literature reviews, in vivo studies that used fibrin glue mixed with various bone substitutes or fibrin glue loaded with growth factors were excluded. Nevertheless, studies that used scaffold containing MSCs in fibrin glue, alone or combined with only a single bone substitute, were considered within the eligibility criteria.

The selected studies were accurately analyzed according to the eligibility criteria in order to minimize possible bias, excluding studies that used fibrin glue mixed with more than one bone substitute or carried with growth factors.

## 3. Results

The electronic bibliographic search found 80 articles in the PubMed/MEDLINE database, of which 63 were excluded because they were outside the eligibility criteria. In the LILACS and Embase databases, we found 79 and 34 articles, respectively, which were not included in this review due to duplicity or because they were outside the eligibility criteria. After analyzing the abstracts of the articles, we selected 12 preclinical and 5 clinical studies from the PubMed/MEDLINE to compose this review (Figure 1). Table 1 and Table 2 present the main outcomes of preclinical and clinical studies that compile this review, respectively.

In preclinical studies, fibrin glue loaded with MSCs was employed to regenerate bone defects in the femur [31,32,34,42], calvaria [33,37,39], maxilla and mandible [35,40,41], as well as to regenerate the cartilage of the knees [36,38]. Most studies used allogeneic MSCs, except for a few studies that opted for autologous MSCs [35,37,39]. In these studies, MSCs were harvested from several sources, such as bone marrow (BM-MSCs) [31,32,34,39,41,42], adipose tissue (AD-MSCs) [33,37,38,40], skin (SD-MSCs) [35] and menstrual blood (MenSCs) [36]. Computed tomography, histological and radiographic analysis were the main methods used to analyze the effect of treatments. In general, in vivo studies have shown that fibrin glue associated with MSCs significantly favored bone regeneration compared to the isolated use of fibrin glue [34,36,41,42].

In the study by Han et al. (2014), bone formation was favored by the use of fibrin glue and MSCs associated with decalcified bone matrix. In contrast, in the study by Cassaro et al. (2019), the use of scaffold containing only fibrin glue and MSCs exhibited higher bone formation than this scaffold associated with biphasic calcium phosphate. In other studies, fibrin glue loaded with MSCs showed a higher regenerative potential than the use of biphasic calcium phosphate with MSCs [39] or similar effect compared to autologous bone graft [40]. Studies that tested fibrin glue mixed with demineralized bone matrix and loaded with MSCs differentiated in osteogenic lineage showed better regenerative potential than the use of this scaffold loaded with undifferentiated MSCs [37]. On the other hand, the use of fibrin glue carried with MSCs showed significant regenerative potential to regenerate bone defects in ovariectomized (OVX) and non-ovariectomized rats, regardless of the stage of differentiation of MSCs [42].

Most of the clinical studies included in this review used fibrin glue loaded with MSCs to treat osteoarthritis and knee cartilage injuries [44,45,46,47]. Only one reported case employed fibrin glue associated with autogenous bone graft and MSCs to treat multiple fractures in the cranial of a 7-year-old girl [43]. All clinical studies applied autologous MSCs to treat the injured tissue. In four studies, MSCs were harvested from fat tissue of the gluteo, except for the study by Haleem et al. (2010) who used MSCs from the iliac crest bone marrow. In most studies, the effect of treatments was analyzed by magnetic resonance imaging, computed tomography, radiographic and ultrasound analysis. The results showed that fibrin glue loaded with MSCs showed considerable potential to regenerate the cartilaginous tissue of the joint, with significant improvement in painful symptoms and in the clinical parameters [44,45,46,47]. In the study by Lendeckel et al. (2004), the bone graft associated with fibrin glue and MSCs also promoted the formation of new bone with near complete cranial continuity after 3 months, without postoperative complications.

## 4. Discussion

This systematic review included preclinical and clinical studies that investigated the regenerative potential of fibrin glue loaded with MSCs to treat bone defects or fractures and cartilage injuries. Some of these studies evaluated the effect of fibrin glue loaded with MSCs, alone or mixed with bone substitute. Other studies have analyzed the effect of fibrin glue loaded with MSCs versus the use of bone substitute. Regardless of the experimental design, all studies showed that fibrin glue associated with MSCs had significant potential to regenerate the injured tissue.

All preclinical studies that compared the regenerative potential of fibrin glue, isolated or loaded with MSCs, showed that the regeneration of bone and cartilage defects was significantly more pronounced in the groups treated with MSCs [34,36,41,42]. In fact, although fibrin glue constitutes an active structure for extracellular matrix biosynthesis and has interaction sites to different growth factors and biomolecules, the presence of this fibrin matrix, by itself, is not enough to regenerate the injured tissue [34,37,39,40,41]. Studies that used animal models showed that the ectopic application of fibrin glue loaded with BM-MSCs promoted bone formation at the implantation site [29,30], with evident expression of osteogenic markers, such as *osteocalcin* and *type I collagen* [30]. In contrast, no formation of mineralized tissue was observed in the groups that received the implant of MSCs or fibrin glue alone [30]. Likewise, a study that used implant of fibrin glue loaded with AD-MSCs found greater ectopic formation of cartilaginous tissue and evident expression of chondrogenic markers, such as cartilage-specific gene called *aggrecan*, *type II collagen* and *SOX-9*, compared to the groups that received only MSCs or fibrin glue [48].

**Figure 4:**
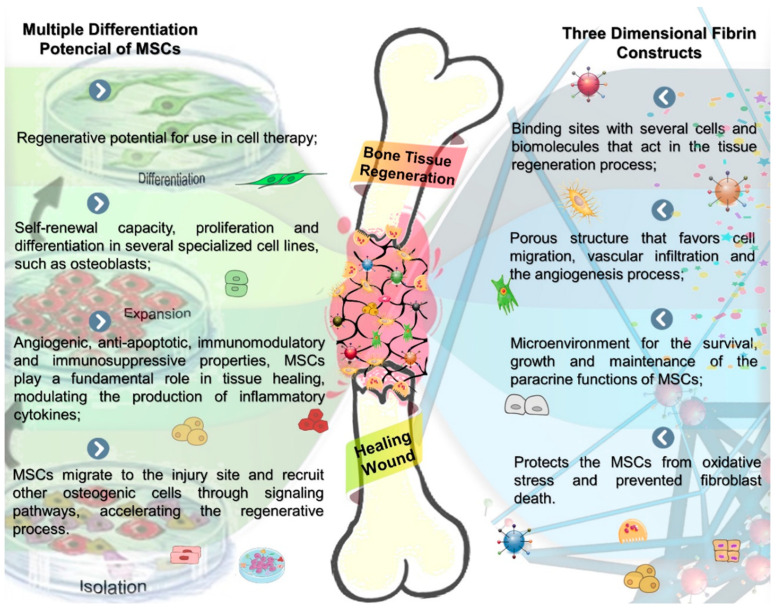
Schematic illustration of the tissue engineering process steps shows the properties of MSCs and fibrin scaffolds, suggesting a synergistic effect on bone formation resulting from a specialized tissue construction for wound healing, see in the center of the figure in a bone defect. Bone fracture healing steps: a spatiotemporal cascade of growth factors regulates bone regeneration during fracture repair. In the initial phases of this process, there is the formation of a blood hematoma incorporating fibrin scaffold with MSCs, attracting cell migration, bioadhesiveness of active molecules and proteins intrinsically related to the bone regeneration process, constituting a favorable microenvironment for osteoid matrix deposition.

However, in association with MSCs, the use of fibrin glue has shown promising results in the field of regenerative medicine [49,50,51,52,53]. Due to their osteoinductive, angiogenic and immunomodulatory properties, MSCs favor tissue regeneration, modulating the production of inflammatory cytokines, through local or paracrine mechanisms [7,8,9,10]. Fibrin glue contributes to this process by favoring the adhesion, proliferation, differentiation and migration of MSCs [13,14,23]. In addition, fibrin glue facilitates vascular infiltration and angiogenesis in the lesion (Figure 4) [13,14,23]. These vascular processes are indispensable for tissue regeneration, as they provide oxygen, nutrients and biomolecules necessary to maintain tissue vitality. A study that evaluated the angiogenic potential of fibrin glue loaded with MSCs and implanted in the subcutaneous region of mice, found a significant increase in vascularization and a decrease in the thickness of the repair tissue at the implant site [54].

One of the disadvantages of fibrin sealants is the low mechanical resistance, which can be improved by the association with a bone substitute [19,25]. While the bone substitute improves the mechanical properties of the scaffold, fibrin glue facilitates the fixation of the graft and promotes better adhesion of the MSCs at the injury site [31,32,33,35,37,38,43]. Considering these issues, some preclinical studies included in this review evaluated the effect of fibrin glue loaded with MSCs and mixed with bone substitute, such as demineralized bone [32,35,37] and *type II collagen* hydrogel [38]. In all these studies, the presence of MSCs also favored the healing of injured tissue [32,35,37,38]. Thus, the use of fibrin glue associated with demineralized bone and loaded with MSCs had a better potential to regenerate bone defects than the use of scaffold without cells [32], [35]. Likewise, *type II collagen* hydrogel and fibrin sealant loaded with AD-MSCs induced greater repair of rabbits chondral lesions and better cell organization and alignment of *collagen* fibers compared to the use of scaffold without MSCs [38]. Additionally, Lazarini et al. (2017) found the presence of chondrocyte-like cells in the deeper areas of the newly formed tissue in defects treated with scaffold containing AD-MSCs, suggesting that a more advanced stage of tissue regeneration may have been achieved by treatment with MSCs.

The studies in this review that investigated the regenerative potential of fibrin glue loaded with MSCs, isolated or in combination with bone substitute, obtained different outcomes [31,33]. In the study by Han et al. (2014), the implantation of scaffold containing fibrin glue, AD-MSCs and decalcified bone matrix (DBM) presented better bone formation in rabbits cranial defects compared to the use of scaffold without DBM. On the other hand, in the study by Cassaro et al. (2019), scaffold containing fibrin glue and BM-MSCs exhibited significantly higher bone formation in rats femur defects than the use of that scaffold associated with biphasic calcium phosphate. The different bone substitutes and the peculiar characteristics of the MSCs harvested from different sources may have influenced the outcomes of the studies by Han et al. (2014) and Cassaro et al. (2019). Despite these differences, all the preclinical studies included in this review showed that fibrin glue loaded with MSCs showed a regenerative potential considerably higher than the use of fibrin glue alone, regardless of the origin of the MSCs.

The preclinical studies that compared the effect of fibrin glue versus some bone substitute showed that fibrin glue loaded with MSCs had a similar [40] or superior regenerative potential [39] to the group treated with bone substitute. Thus, fibrin glue loaded with MSCs accelerated the osteogenesis in rabbits mandibular defects and showed regenerative potential similar to the group treated only with autologous bone graft, with no significant difference between these groups [40]. This data showed that fibrin glue loaded with MSCs can be an interesting option for non-invasive treatment in maxillofacial surgery [40].

In the study by Lee et al. (2008), the use of fibrin glue loaded with MSCs resulted in earlier and more mature new bone formation in rabbits calvarial defects when compared to the use of biphasic calcium phosphate associated with MSCs. In this study, biphasic calcium phosphate was still found at the defect site after 3 months and a foreign body reaction with accumulation of lymphocytes, plasma cells and histiocytes was associated with the use of this bone substitute. In contrast, residual fibrin glue still remained 2 months after implantation and no granulation tissue reaction was found [39]. In the study by Cassaro et al. (2019), the presence of fibrin glue was not detected at the defect site after 30 days, suggesting that the reabsorption of the fibrin matrix occurred simultaneously with the formation of new bone. In many situations, the rate of degradation of fibrin glue can represent an advantage over bone substitutes of slow degradation.

Regarding the cell differentiation, two studies included in this review compared the effect of fibrin glue loaded with MSCs, undifferentiated or differentiated in osteogenic lineage [37,42]. Kim et al. (2012) reported that the use of fibrin glue (FG) mixed with demineralized bone matrix (DBX) and loaded with MSCs differentiated in osteogenic lineage (iMSCs) showed the best potential to regenerate critical calvarial bone defects in rats, compared to the use of scaffold with undifferentiated MSCs. The mean radiodensity of the FG, FG + DBX, FG + DBX + MSCs and FG + DBX + iMSCs groups was 16.78%, 39.94%, 25.58% and 51.31%, respectively [37]. On the other hand, Orsi et al. (2017) investigated the regenerative potential of fibrin glue loaded with MSCs to regenerate femur defects in ovariectomized (OVX) and non-ovariectomized (NOVX) rats. In both OVX and NOVX groups, fibrin glue with undifferentiated or differentiated MSCs showed considerable regenerative potential and no morphological differences in the femurs of the OVX and NOVX rats were observed after the surgery.

Corroborating with preclinical studies, the regenerative potential of fibrin glue with MSCs has also been demonstrated in clinical studies [44,45,46,47]. Clinical trials have shown that fibrin glue and MSCs can be considered a promising strategy for treating joint injuries. In the study by Haleem et al. (2010), 3 of the 5 treated patients showed complete defect regeneration and complete surface congruity with native cartilage. The partial regeneration of the defect was found only in 2 patients who had more advanced joint degenerations in the preoperative. Even so, treatment with fibrin glue loaded with MSCs provided significant relief from painful symptoms and enabled patients to return to their daily activities. In addition, no cases of infection or postoperative complications were reported in this study (Haleem et al., 2010). In agreement with these findings, later studies that injected fibrin glue with MSCs to treat patients with knee osteoarthritis and symptomatic knee cartilage defects on the femoral condyle, reported that fibrin glue has proven to be an effective scaffold in MSC implantation to treat cartilage injuries [45,46,47].

In these studies, the interactions between cells, matrix components and growth factors present in the microenvironment favored the chondrogenic differentiation of MSCs, stimulating the formation of cartilage at the injury site [55]. In many clinical cases, the use of fibrin glue can be advantageous over other biomaterials because it is possible to use autologous scaffolds. In addition, the components of the fibrin glue can be injected into the lesion site, completely filling the defect, with subsequent gel solidification [19]. The regenerative potential of fibrin glue loaded with MSCs was also demonstrated in a report case that implanted cancellous bone associated with fibrin glue and MSCs in multifragment cranial fracture in a 7-years-old girl [43]. In this study, fibrin glue was used to maintain MSCs at the site of the bone defect [19], and the results showed new bone formation in the cranial with near complete cranial continuity after 3 months of postoperative, without uneventful or neurological deficits. However, the authors were unable to determine how much of the effect was due to the FG + MSCs or to the bone graft [43].

The studies included in this review evaluated the outcome of treatments through various methods, which involve histological and biochemical analysis, immunofluorescence assays, biomechanical tests, scanning electron microscopy, magnetic resonance imaging, microcomputed tomography, radiography and radiodensitometry. Most of these studies used a combination of methods to analyze bone or cartilage regeneration, except for the study by Kang et al. (2010) and Lazarini et al. (2017), who evaluated the effect of treatments only by histological analysis. In contrast with other authors, the study by Kim et al. (2015), evaluated the regenerative potential of treatments by applying three different indices (International Knee Documentation Committee score, Tegner Activity scale and International Cartilage Repair Society grade). Overall, the available techniques are useful tools to assess the effect of drugs or therapeutic interventions on injured tissue. The use of more than one technique provides more comprehensive data about the neoformed tissue.

With respect to bone, these analyzes are able to detect the qualitative and quantitative changes that occur over time, considering that bone is a metabolically active tissue. In this way, histological analysis and the use of fluorochromes are useful to assess extracellular matrix deposition and mineralization. Additionally, biochemical analyzes allow the detection of biomarkers involved in bone metabolism and histomorphometry is a valuable technique to assess cell activity [56,57]. Other techniques have been extensively used to assess bone tissue density and microarchitecture, such as densitometry and microtomography, respectively. Magnetic resonance imaging is a non-invasive technique that assesses bone architecture with high resolution and specificity [58,59]. Biomechanical tests provide additional information about the quality of newly formed bone, such as fracture strength. Thus, most of the studies included in this review presented consistent data about the effect of treatments on bone defects or cartilage lesions.

The use of fibrin glue mixed with biomaterials or bone substitutes has been an option for the clinical treatment of multiple defects or extensive lesions, considering the low mechanical strength and the biological degradation rate of fibrin glue [19,25]. With regard to cells, most of the clinical studies included in this review employed autologous MSCs harvested from adipose tissue. Regardless of possible differences in the molecular profile of MSCs from different sources, adipose tissue has been preferentially chosen in clinical interventions because it constitutes an abundant source of MSCs, which can be obtained by a less invasive procedure [5]. Regarding the cultivation of MSCs, some issues are currently being discussed. 2-D cultivation does not provide a three-dimensional microenvironment, which reflects cell-cell and cell-matrix interactions. Furthermore, 2-D culture with multiple passages can affect the differentiation potential and accelerate cell senescence. Recently, some 3-D cultivation methods using biomaterials have been proposed to mimic the natural niche of cells. However, in both cases, challenges still exist to maintain the genetic stability and differentiation potential of the cells [1].

Several issues can influence the outcomes of the studies, such as the experimental design, the characteristics of the lesion and the population of the study, the composition of the scaffold and the concentration of the fibrin glue components. Variations in *thrombin* and *fibrinogen* concentrations can affect the porosity of the fibrin matrix, as well as cell proliferation, differentiation and migration in the scaffold [12,18,19,23,25]. However, all studies in this review that tested fibrin glue loaded with MSCs obtained satisfactory results in terms of tissue regeneration and showed that the fibrin glue can be used as an effective cell carrier for the lesion site.

## 5. Conclusions

Fibrin glue is a natural polymer that has proven to be an effective delivery system or scaffold matrix. In cell therapy strategies, the association of fibrin glue with MSCs has shown promising results in several segments of regenerative medicine. Fibrin glue favors the adhesion, proliferation and differentiation of MSCs, which have important biological functions in the injury site. In addition to acting as a sealing and hemostatic agent, fibrin glue favors angiogenesis and deposition of extracellular matrix due to interactions with various cells and growth factors.

This systematic review showed clear evidence, based on preclinical and clinical studies, that fibrin glue loaded with MSCs, alone or in association with bone substitute, has the potential to regenerate bone or cartilage lesions. However, further studies should be conducted to assess the clinical therapeutic effects of this strategy on bone tissue engineering.

## Figures and Tables

**Table 1:** Preclinical studies selected according to eligibility criteria.

Reference	Stem Cells Source	Treatment Groups Delivery System	Intervention Implantation Site	Analysis	Main Outcomes Conclusions
Cassaro et al., 2019 [31]	Allogeneic rats BM-MSCs (femurs/tibias)	G1: No filling G2: Biphasic calcium phosphate (BCP) G3: Fibrin biopolymer (FBP) + BCP G4: FBP + MSCs G5: FBP + BCP + MSCs	Implantation of the scaffold in a 5 mm bone defect in the right femur of the rats (*n* = 8). Analyzes were performed after 30 and 60 days of the procedures.	Computed tomography, scanning electron microscopy and histological analysis.	All groups exhibited bone matrix formation, with significantly higher bone formation in FBP + MSCs. FBP proved to be an excellent scaffold for bone repair therapies.
Chen et al., 2014 [32]	Allogeneic rabbits BM-MSCs (femurs)	G1: Decalcified bone matrix (DBM) + fibrin gel (FG) + MSCs G2: DBM + FG G3: No filling	Implantation of the scaffold in a 10 mm bone defect in the left femur of the rabbits (*n* = 10). Analyzes were performed after 12 weeks of the procedures.	Serum proteomics (2D-PAGE and MALDI-TOF-TOF-MS), hematoxylin–eosin (HE) staining, ALP staining and osteopontin immunofluorescence detection.	DBM + FG + MSCs exhibited better bone regeneration than other groups. The combination of DBM + FG + MSCs can result in successful bone formation and can be used as a scaffold for bone tissue engineering.
Han et al., 2014 [33]	Human AD-MSCs (human abdominal fat)	G1: Decalcified bone matrix (DBM) + fibrin glue (FG) G2: MSCs + FG G3: MSCs + DBM + FG G4: No filling	Implantation of the scaffold in a 10 mm cranial defects in rabbits (*n* = 5). Analyzes were performed after 6 weeks of the procedures.	Computed tomography and histological analysis.	MSCs + DBM + FG presented better bone formation than others groups. Scaffold containing MSCs could be helpful in the correction of extensive bone defects.
Hao et al., 2014 [34]	Allogeneic rats BM-MSCs (femurs/tibias)	G1: control group G2: atrophic nonunion Group—fibrin glue (FG) G3: experimental group (MSCs + FG)	Injection of FG and MSCs + FG into the osteotomized right rat femur (with a length of 1 mm) of G2 and G3, respectively (*n* = 12). Analyzes were performed after 8 weeks of the procedures.	Radiographic and histological analysis. Biomechanical test.	MSCs + FG presented complete bony bridging of the osteotomy gap, with the formation of plenty of woven bone. Local injection of MSCs seeded fibrin glue promoted atrophic nonunion repair.
Kang et al., 2010, [35]	Autogenous pigs skin-derived MSCs (SD-MSCs) (ears)	G1: Demineralized bone (DMB) + fibrin glue (FG) + MSCs G2: Demineralized bone (DMB) + fibrin glue (FG)	Implantation of the scaffold in a lateral window (1 cm) in maxillary sinus of 4 pigs. In each pig, one maxillary sinus received only the scaffold and the other sinus received the scaffold with MSCs. Analyzes were performed after 2 and 4 weeks of the procedures.	Histological analysis.	Trabecular bone formation were more pronounced in DMB + FG + MSCs, Autogenous MSCs grafting with a DMB + FG scaffold can serve as a predictable alternative to bone grafting in the maxillary sinus floor.
Khanmoha-mmadi et al., 2019 [36]	Human menstrual blood-derived stem cells (MenSCs)	G1: Right defect: fibrin glue (FG) + MenSCs Left defect: FG G2: Left defect: No filling	Implantation of the scaffold in a knees osteochondral defects (3 × 4 mm^2^) of the rabbits (*n* = 12). Analyzes were performed after 3 and 6 months of the procedures.	Gross morphological and histological analysis.	The most regenerated tissue in FG + MenSCs was similar to hyaline cartilage and it was higher than other experimental groups. MenSCs encapsulated in FG was more effective in defect repair compared to FG alone.
Kim et al., 2012 [37]	Autogenous Rats AD-MSCs (inguinal adipose tissue)	G1: Fibrin glue (FG) G2: FG + Demineralized Bone Matrix (DBX) G3: FG + DBX + MSCs G4: FG + DBX + iMSCs (osteogenic induced)	Implantation of the scaffold in a 8 mm critical calvarial bone defect in rats (*n* = 10). Analyzes were performed after 8 weeks of the procedures.	Radiographic, histological and radiodensitometric analysis.	The mean radiodensity of the FG, FG + DBX, FG + DBX + MSCs and FG + DBX + iMSCs groups was 16.78%, 39.94%, 25.58% and 51.31%, respectively. FG + DBX + iMSCs group showed the better potential to regenerate bone defects.
Lazarini et al., 2017 [38]	Human AD-MSCs (abdominal liposuction)	Type II *collagen* hydrogel and fibrin sealant implant with (right knee) or without AD-MSCs (left knee)	Implantation of the scaffold in a 3 mm knees defects in 4 rabbits. Analyzes were performed after 12 weeks of the procedures.	Histological analysis.	Scaffold containing MSCs induced greater repair of chondral lesions and better cell organization and alignment of *collagen* fibers compared to the isolated use of the scaffold, being effective for articular cartilage repair.
Lee et al., 2008 [39]	Autogenous Rabbits BM-MSCs (iliac bone)	Group 1 (15 rabbits): Defect 1: MSCs + autologous fibrin glue (AFG) Defect 2: MSCs + macroporous biphasic calcium phosphate (MBCP) Defect 3: No filling Group 2 (3 rabbits): Defect 1: AFG Defect 2: MBCP Defect 3: No filling	Implantation of the scaffold in a 6 mm cranial defects (3 defects/rabbit). Analyzes were performed after 1, 2 and 3 months of the procedures.	Radiographic and histological analysis.	MSCs + AFG induced more bone formation 2 months post operation and more mature bone was found 3 months post operation compared with the other groups. MSCs + AFG resulted in earlier and more mature new bone formation.
Mehrabani et al., 2018 [40]	Allogeneic Rabbits AD-MSCs (subcutaneous adipose tissue)	G1: (*n* = 10) Right defect: autologous fibrin glue + MSCs Left defect: No filling (control) G2: (*n* = 10) Right defect: autologous fibrin glue Left defect: autologous bone graft (iliac crest)	Implantation of the scaffold in a bilateral 1.5 × 0.5 cm uni-cortical mandibular osteotomies in 20 rabbits. Analyzes were performed after 28 and 56 days of the procedures.	Computed tomography and histopathological analysis	There was accelerated osteogenesis in the treated defects, with better bone formation in FG + MSCs and autologous bone graft groups, which showed a significant and similar increase in the thickness of new cortical bone. FG + MSCs presented a remarkable reconstruction of cortical bone.
Zhang et al., 2012 [41]	Allogeneic rats BM-MSCs (femurs)	G1: blank (no filling) G2: fibrin glue (FG) G3: FG + MSCs	Implantation of the scaffold in a bilateral maxillar defects (3 mm) in rats (*n* = 5). Analyzes were performed after 6 weeks of the procedures.	Histological analysis and micro-CT.	The amount of new bone formed in the FG + MSCs was significantly greater than the others groups. The strategy of combing MSCs with FG is effective in the repair of alveolar bone defects.
Orsi et al., 2017 [42]	Allogeneic rats BM-MSCs (femurs)	Non-ovariectomized (NOVX): G1: Fibrin sealant (FS) G2: FS + MSCs G3: FS + iMSCs (differentiated in osteogenic lineage) G4: No filling G5: No injury Ovariectomized (OVX): G1: FS G2: FS + MSCs G3: FS + iMSCs G4: No filling G5: No injury	Implantation of the scaffold in a femur defects (5 mm) in rats (*n* = 4). Analyzes were performed after 14 and 28 days of the procedures.	Microcomputed tomography, biochemical analysis, radiographic and histology analysis, scanning electron microscopy.	After 14 days, in both the OVX and NOVX animals, FS + MSCs and FS + iMSCs showed a higher formation of bone cells in relation to the control group. Bone neoformation was observed in all treated and control groups. No morphological differences in the femurs of the NOVX and OVX animals were observed after the surgery.

**Table 2:** Clinical studies selected according to eligibility criteria.

Reference	Stem Cells Source	Treatment Groups Delivery System	Intervention Implantation Site	Analysis	Main Outcomes Conclusions
Lendeckel et al., 2004 [43]	Autologous human AD-MSCs (gluteal area)	Autologous cancellous bone (iliac crest) + autologous fibrin glue (FG) + autologous MSCs	Implantation of bone + FG + MSCs in multifragment cranial fracture in a 7-years-old girl. Analysis were performed after 3 months of the surgery.	Computed tomography (CT) and ultrasound analysis.	Postoperative healing was uneventful and without neurological deficits. CT-scans 3 months post-operatively showed new bone formation and near complete cranial continuity. There is no way to determine how much of the effect was due to the grafted bone or the FG + MSCs.
Haleem et al., 2010 [44]	Autologous human BM-MSCs (iliac crest)	platelet-rich fibrin glue (PR-FG) + MSCs	Implantation of the scaffold in 5 patients with cartilage lesion in the femoral condyle. Analyzes were performed after 6 and 12 months of the procedures.	Radiographic and magnetic resonance imaging analysis.	Complete defect fill and complete surface congruity with native cartilage was found in 3 patients, while 2 patients presented incomplete congruity. BM-MSCs transplantation on PR-FG as a scaffold may be an effective approach to promote the repair of articular cartilage defects of the knee in human patients.
Kim et al., 2015 [45]	Autologous human AD-MSCs (buttock liposuction)	G1: MSCs G2: MSCs + fibrin glue (FG)	Injection of MSCs (*n* = 37) or scaffold containing MSCs (*n* = 17) in patients with osteoarthritis in the knees. Analyzes were performed after approximately 29 months of the procedures.	Evaluation by International Knee Documentation Committee (IKDC) score, Tegner Activity scale and International Cartilage Repair Society (ICRS) grade.	There were no significant differences in outcome scores between groups However, at second-look arthroscopy, there were better ICRS grades in G2 (23% of lesions in G1 and 58% in G2 achieved grade I and II). Fibrin glue has proven to be an effective scaffold in MSC implantation for osteoarthritis knees treatment.
Koh et al., 2016 [46]	Autologous human AD-MSCs (buttock liposuction)	G1: microfracture (MFX) + fibrin glue (FG) + MSCs G2: MFX alone	Injection of scaffoldin patients with symptomatic knee cartilage defects (> 3 cm^2^) on the femoral condyle (*n* = 40). Analysis were performed after 24 months of the procedures.	Evaluation by magnetic resonance imaging, Lysholm score, Knee Injury and Osteoarthritis Outcome Score (KOOS) and a 10-point visual analog scale for pain.	G1 included 26 patients (65%) who had complete cartilage coverage of the lesion at follow-up compared with 18 patients (45%) in G2. The improvements in the mean KOOS pain and symptom subscores were significantly greater at follow-up in G1 than in G2. Compared with MFX alone, MFX + FG + MSCs provided an improved radiologic appearance of lesions and KOOS pain/symptom subscore improvements.
Kim et al., 2017 [47]	Autologous human AD-MSCs (buttock liposuction)	G1: arthroscopic rotator cuff repair G2: arthroscopic rotator cuff repair + injection of AD-MSCs in fibrin glue (FG)	Injection of scaffols containing MSCs in patients with osteoarthritis in the knees (*n* = 35/per group). Analysis were performed after approximately 28 months of the procedures.	Evaluation by magnetic resonance imaging (MRI), visual analog scale (VAS), range of motion (ROM), functional measures of Constant score and University of California, Los Angeles (UCLA) shoulder rating scale.	MRI indicated a retear rate of 28.5% in G1 and 14.3% in G2. There was no significant difference between groups in the other parameters analyzed. The injection of FG + MSCs during rotator cuff repair could significantly improve structural outcomes in terms of the retear rate.

## Data Availability

Not applicable.
